# A Multi-Model Image Enhancement and Tailored U-Net Architecture for Robust Diabetic Retinopathy Grading

**DOI:** 10.3390/diagnostics15182355

**Published:** 2025-09-17

**Authors:** Archana Singh, Sushma Jain, Vinay Arora

**Affiliations:** Department of Computer Science and Engineering, Thapar Institute of Engineering and Technology, Patiala 147004, Punjab, India; sjain@thapar.edu (S.J.); vinay.arora@thapar.edu (V.A.)

**Keywords:** biomedical image processing, data augmentation, deep learning, diabetic retinopathy, image classification, U-Net

## Abstract

**Background:** Diabetic retinopathy (DR) is a leading cause of preventable vision impairment in individuals with diabetes. Early detection is essential, yet often hindered by subtle disease progression and reliance on manual expert screening. This study introduces an AI-based framework designed to achieve robust multiclass DR classification from retinal fundus images, addressing the challenges of early diagnosis and fine-grained lesion discrimination. **Methods:** The framework incorporates preprocessing steps such as pixel intensity normalization and geometric correction. A Hybrid Local-Global Retina Super-Resolution (HLG-RetinaSR) module is developed, combining deformable convolutional networks for local lesion enhancement with vision transformers for global contextual representation. Classification is performed using a hierarchical approach that integrates three models: a Convolutional Neural Network (CNN), DenseNet-121, and a custom multi-branch RefineNet-U architecture. **Results:** Experimental evaluation demonstrates that the combined HLG-RetinaSR and RefineNet-U approach consistently achieves precision, recall, F1-score, and accuracy values exceeding 99% across all DR severity levels. The system effectively emphasizes vascular abnormalities while suppressing background noise, surpassing existing state-of-the-art methods in accuracy and robustness. **Conclusions:** The proposed hybrid pipeline delivers a scalable, interpretable, and clinically relevant solution for DR screening. By improving diagnostic reliability and supporting early intervention, the system holds strong potential to assist ophthalmologists in reducing preventable vision loss.

## 1. Introduction

**Figure 1:**
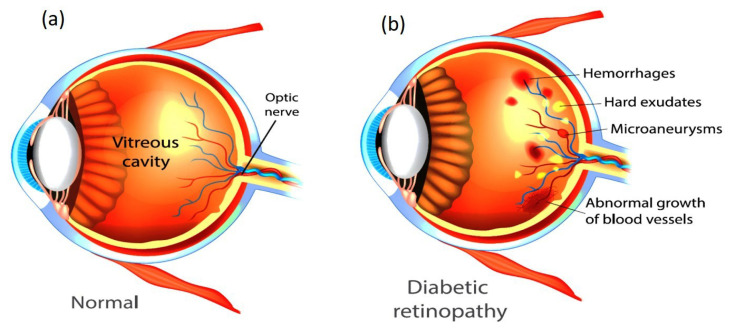
Healthy eye (**a**) vs. diabetic retinopathy-affected eye (**b**).

The human retina is vital for vision, and its health is crucial for preserving total ocular function. The delicate tissue layer in the retina comprises photoreceptor cells that capture light and transmit visual signals to the brain. Diabetic retinopathy (DR) is a prevalent consequence of diabetes and affects the retinal artery and blood vessels due to prolonged elevated blood glucose levels that may lead to swelling, leakage, or complete occlusion of retinal blood vessels, resulting in compromised vision [1]. As DR progresses, aberrant neovascularization may occur on the retinal surface, resulting in further problems such as retinal detachment or bleeding [2]. Figure 1 illustrates the visual comparison between a healthy eye and an eye affected by DR. Early detection of DR is essential to avert visual impairment [3]. Retinal images are valuable for detecting DR and assessing its severity [4,5]. By examining these images, experts may evaluate the condition of blood vessels and identify abnormalities such as microaneurysms, hemorrhages, or neovascularization, which are key indicators of disease progression [6]. Retinal imaging and automated classification models facilitate the monitoring of retinal health and provide essential insights for the early diagnosis and treatment of DR [7]. These technologies enhance the ability to detect and manage the condition effectively through the following key aspects:

### 1.1. Retinal Imaging as a Diagnostic Tool

Retinal imaging plays a vital role in assessing the health of the retina and its associated blood vessels in individuals with DR [8]. High-resolution imaging modalities, such as fundus photography and Optical Coherence Tomography (OCT), allow clinicians to capture detailed views of retinal structures in a non-invasive and cost-effective manner. These techniques help to detect early signs of DR, including microaneurysms, hemorrhages, and exudates, which are key markers for grading disease severity. Image processing methods further enhance these diagnostic images by highlighting relevant features and facilitating the identification of subtle pathological changes. By enabling large-scale screening with minimal discomfort, retinal imaging supports timely diagnosis and monitoring, making it an essential tool in DR diagnosis.

### 1.2. Role of Artificial Intelligence in DR Diagnosis

The adoption of AI and machine learning has transformed the way DR is detected and classified [9]. Advanced AI models, especially deep learning approaches, excel at analyzing complex retinal images to identify disease-specific patterns that might be overlooked by manual assessment [10]. Convolutional Neural Networks (CNNs), in particular, have demonstrated strong performance in detecting retinal lesions, abnormal vessel growth, and other critical indicators of DR progression [11]. Beyond CNNs, techniques such as deformable convolutional networks [12,13] and vision transformers [14] further improve feature extraction by capturing both fine local details and broader contextual information within retinal scans. These models can process data from fundus photographs and OCT images to distinguish healthy retinal tissue from disease-affected regions. Integrating AI into screening workflows helps automate grading, reduce the burden on specialists, and supports early intervention, ultimately improving patient outcomes.

### 1.3. Enhancement and Classification of Retinal Images

The enhancement and classification of retinal images are critical in medical imaging, particularly for diagnosing and monitoring DR and other retinal diseases. Image enhancement techniques are crucial in enhancing the visibility of key features in fundus images. Enhancing contrast and reducing noise allows automated systems to distinguish between healthy and pathological regions of the retina better. Several image enhancement methods have been introduced in the literature to address the challenges associated with low-contrast retinal images. These include the following:

- **Histogram Equalization**—a widely used technique that redistributes the intensity values of an image to enhance contrast, making subtle details more prominent [15].
- **Adaptive Histogram Equalization**—an extension of HE that applies local contrast enhancement by computing histograms for small regions of the image, thereby improving visibility in darker areas [16].
- **Contrast-Limited Adaptive Histogram Equalization**—a refined version of AHE that limits contrast amplification in high-contrast regions to prevent over-enhancement and noise amplification [17].
- **Enhanced Sub-Image Histogram Equalization**—a technique that divides the image into sub-images and applies histogram equalization separately to each region, preserving finer details [18].

Various machine learning and deep learning techniques have been employed in DR detection to achieve precise classification [19,20]. Traditional machine learning algorithms such as SVMs [21], RFs [22], K-Nearest Neighbors (KNN) [23], Decision Trees (DT) [24], and Naïve Bayes (NB) [25] have been explored for classifying retinal images based on handcrafted features like texture, color, and blood vessel morphology. Feature extraction methods such as Principal Component Analysis (PCA) [26], Histogram of Oriented Gradients (HOG), and Local Binary Patterns (LBP) have also been used to improve classification performance in conventional machine learning models. With advancements in deep learning, Convolutional Neural Networks (CNNs) have demonstrated superior performance in automatically extracting relevant features from retinal images, eliminating the need for manual feature engineering. Various architectures such as ResNet, VGGNet, InceptionNet, DenseNet, and EfficientNet have been widely adopted for diabetic retinopathy (DR) classification, achieving high precision in detecting different severity levels. Moreover, transfer learning techniques utilizing pre-trained models like InceptionV3, Xception, and MobileNet have further enhanced classification outcomes, especially when working with limited medical imaging datasets.

Building on these developments, the present study addresses class imbalance and feature extraction challenges in DR datasets by integrating adaptive loss functions, improved gradient flow, and a comprehensive image preprocessing pipeline. This includes deformable convolutions and vision transformers to boost image quality and contextual understanding. To further refine classification performance, this study implements a U-shaped architecture with skip connections for effective feature fusion and introduces RefineNet-U, an enhanced U-Net-based model specifically designed to improve feature representation and classification accuracy in multi-stage DR detection.

### 1.4. Key Contributions

The key contributions of this work are summarized as follows:

- **Development of the HLG-RetinaSR Module:** A Hybrid Local–Global Super-Resolution (HLG-RetinaSR) module is proposed to enhance retinal image quality. By integrating deformable convolutional layers and a vision transformer, the module effectively isolates and crops local lesion areas while suppressing irrelevant background regions. This targeted enhancement reduces noise interference and significantly boosts classification accuracy.
- **Feature Fusion Strategy:** A comprehensive feature fusion mechanism is implemented through a U-shaped structure with skip connections for seamless integration of fine-grained details and high-level semantic features. This design preserves critical information during downsampling and enhances classification accuracy upon upsampling.
- **Proposed RefineNet-U Model:** A novel multi-branch fine-grained classification architecture, RefineNet-U, is introduced based on the U-Net backbone. This model is specifically designed to improve the accuracy of DR grade classification by capturing hierarchical lesion representations. U-Net offers several distinct advantages:
  - **Precise Lesion Localization:**
    The U-Net structure facilitates the accurate identification of DR lesions, such as microaneurysms and exudates. By effectively pinpointing these abnormalities, clinicians can better determine disease severity and progression.
  - **Context-Aware Feature Extraction:**
    The network’s contracting path employs downsampling to capture high-level contextual information. This ability to learn multiple scales of features ensures that U-Net can discern nuanced details in retinal images, leading to more robust DR classification.
  - **U-shaped Architecture with Skip Connections:**
    U-Net comprises a contracting path for feature extraction and an expanding path for refined localization. Skip connections link matching layers in these paths, seamlessly integrating detailed lower-level features with broader high-level representations. This architecture maintains essential spatial information crucial for detecting small lesions in retinal images.
- **Enhanced Healthcare Management:** The proposed method aids in advancing healthcare management by enabling the early detection and precise classification of DR. This facilitates healthcare practitioners in implementing timely interventions, improving patient care and alleviating the strain on healthcare infrastructure.

This paper is organized into five key sections to ensure a clear and logical presentation of the study. Section 1 outlines the research problem, objectives, and significance, providing the context and motivation for the study. Section 2 critically examines existing research, identifies gaps in current knowledge, and establishes the theoretical framework for this work. Section 3 provides a detailed description of the techniques and approaches employed in the research. Section 4 presents the experimental findings, interprets their implications, and compares them with previous studies. Finally, Section 5 summarizes the key insights derived from the study and highlights the practical applications and future directions of the research.

## 2. Literature Review

Over recent decades, extensive efforts have been made to enhance automated disease classification, particularly for DR. DR classification remains a well-established area in medical imaging, with numerous studies focusing on automated detection and grading techniques. This review highlights key developments and methodologies in DR classification, emphasizing approaches that remain relevant with the evolution of machine and deep learning technologies.

### 2.1. Image Enhancement Approaches

Accurate segmentation and diagnosis of DR hinge on having retinal images that are both clear and uniformly illuminated. Over the years, various image enhancement and preprocessing methodologies have been proposed to address issues such as poor contrast and uneven illumination. HE methods like CLAHE have been widely employed to improve image contrast. Shome et al. (2011) [27] and Liu et al. (2023) [28] demonstrated how CLAHE effectively enhances contrast and mitigates uneven illumination in fundus images. Raj et al. (2022) [29] introduced Deep Learning-based Enhancement with Residual Densely Connected U-Net (RDC-U-Net), leveraging residual dense blocks to enhance local and global image features. This approach has shown superior performance in restoring degraded retinal images. Generative models such as CycleGAN, proposed by You et al. (2020) [30], utilize unsupervised learning to improve retinal image quality. By incorporating convolutional block attention modules, these generative models effectively reduce noise while preserving critical retinal structures. Techniques like median filtering have been extensively employed to refine fundus image clarity through the removal of noise. Raj et al. (2019) [31] demonstrated how such filters enhance image quality, thereby facilitating more precise analysis in subsequent DR detection. Several studies underscore the importance of normalization, illumination correction, and other preprocessing steps (e.g., cropping, resizing) to achieve consistent image quality before classification. Such steps are crucial for improving model robustness, particularly in medical imaging.

### 2.2. Machine Learning Approaches

Before the advent of deep learning, DR detection primarily relied on conventional machine learning models. These methods often involved handcrafted feature extraction techniques, which were then fed into classifiers such as SVMs and RFs. Traditional models depended heavily on manually extracted features such as texture descriptors, color histograms, and morphological features to capture disease-specific patterns. However, these handcrafted features did not always generalize well to diverse datasets. Gayathri et al. (2020) proposed a Multipath CNN (M-CNN) for predicting DR, outperforming previous techniques in classification accuracy on publicly available datasets [32]. While early machine learning approaches demonstrated promising results, reliance on domain expertise for feature engineering limited scalability. Moreover, the performance of these models was highly sensitive to variations in image quality and acquisition conditions. In 2024, Hasan et al. [33] used CNN for classification using images from handwritten MNIST datasets. These datasets are used for both training and testing purposes using CNNs. It shows an accuracy of 98.00%. Images used for training purposes are small and gray-scaled. The computational time for processing these images is very high compared to other normal JPEG images.

### 2.3. Deep Learning Approaches

The emergence of deep learning, especially CNNs, revolutionized DR classification by automating feature extraction. CNNs such as AlexNet, VGG, and ResNet have been successfully fine-tuned on DR datasets, showcasing significant improvements in accuracy [34]. Models like DRNet13 introduced specialized convolutional layers and dropout mechanisms to combat overfitting, achieving notable accuracy (97.00%) in DR detection [35]. Building on these foundational CNNs, advanced strategies now integrate attention mechanisms, specialized loss functions, and multi-scale feature extraction to capture complex retinal abnormalities more effectively. Hybrid deep learning models further push performance boundaries by combining multiple architectures or incorporating GANs. GANs serve as robust tools for augmenting limited datasets, thus improving the generalizability of DR classification models [36,37]. Navaneethan and Devarajan (2024) proposed the MGA-CSG algorithm, a hybrid GAN-based method that employs crossover Grasshopper and Salp Swarm optimizers [38]. This approach achieved an accuracy of 98.08%, outperforming many conventional deep learning models. Other hybrid frameworks combine multiple CNN architectures or incorporate additional modules (e.g., attention blocks) to optimize performance [39]. Some recent works incorporate auxiliary data types or sophisticated attention mechanisms to improve DR diagnosis using Multi-Modal and Attention-Based Networks. Al-Antary et al. (2021) [40] explored the integration of OCT data with fundus images in a multi-scale attention network. This enriched data fusion strategy substantially improved diagnostic accuracy. Minarno et al. (2022) [41] demonstrated how combining preprocessing methods and hyperparameter tuning with EfficientNet-B7 led to a test accuracy of 84.36%. Similarly, Minarno et al. (2024) [42] emphasized the importance of data augmentation through rotations, flips, and cropping to address the class imbalances often encountered in medical imaging.

### 2.4. Research Gap

A comprehensive review of the literature shows that various automated techniques have been developed for detecting and classifying retinal disorders, particularly DR. However, important gaps in this field remain and are discussed below:

- **Inadequate Fine-Grained Lesion Discrimination:** Most existing models fail to distinguish subtle pathological features such as microaneurysms, hard exudates, or early neovascularization with sufficient granularity, especially in early-stage DR. This limits their effectiveness in clinically actionable early diagnosis.
- **Limited Integration of Local–Global Context:** Many DR classification frameworks prioritize either global structural features or local lesion-level information, but rarely fuse both effectively. This results in incomplete representations of disease progression.
- **Suboptimal Image Enhancement Pipelines:** Conventional super-resolution or enhancement methods often overlook task-specific lesion enhancement. They improve general image quality but do not necessarily boost classification performance in clinical contexts.
- **Lack of Adaptivity to Retinal Variability:** Existing models show poor generalization across fundus images with varied acquisition conditions (illumination, resolution, patient demographics). There is a need for adaptive enhancement–classification systems that are robust across datasets.
- **Underutilization of Transformer-Based Architectures:** While transformers have shown promise in medical imaging, their integration with CNNs for joint lesion localization and classification remains underexplored in DR pipelines.

Despite advances in DR classification, several challenges persist, primarily due to variability in retinal image quality. Noise, artifacts, and uneven illumination often degrade model performance and lead to unreliable predictions. These issues necessitate advanced preprocessing to enhance image clarity prior to classification. Additionally, many models exhibit limited generalizability, performing well on specific datasets but poorly on unseen data, underscoring the need for robust cross-dataset adaptability through strategies such as dataset diversification and transfer learning [43]. Another critical barrier is the lack of interpretability in deep learning-based systems, which often function as black boxes, reducing clinician trust and hindering clinical adoption. Moreover, current approaches typically treat image enhancement and classification as separate processes. In contrast, the methodology proposed in this study integrates both stages into a unified pipeline, where enhancement improves lesion visibility and supports more accurate and robust DR classification.

## 3. Proposed Methodology

**Figure 2:**
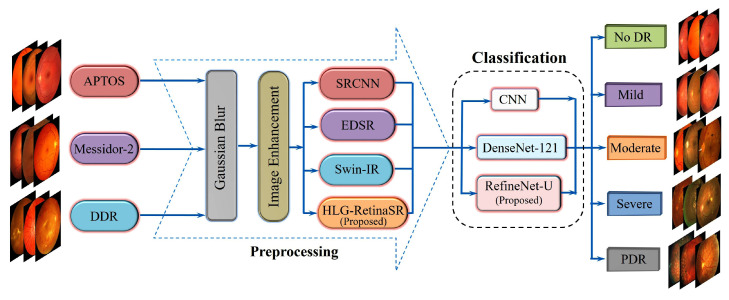
Proposed diabetic retinopathy classification framework.

The proposed approach utilizes a novel deep-learning framework for diagnosing DR by integrating advanced image enhancement and classification models. This section has three fundamental stages: image preprocessing, image enhancement, and classification. During the preprocessing phase, fundus images were first converted into super-resolved grayscale images with four different super-resolution models, namely SRCNN, EDSR, Swin-IR, and a novel approach through the combination of deformable convolution with a vision transformer named HLG-RetinaSR. The enhanced images provide high-quality inputs for the classification phase and were classified using three separate models: CNN, DenseNet-121, and RefineNet-U. This multi-model classification framework facilitates an extensive assessment, including benchmark samples that enhance the classifier’s learning and training process. Figure 2 shows the proposed DR classification framework.

**Figure 3:**
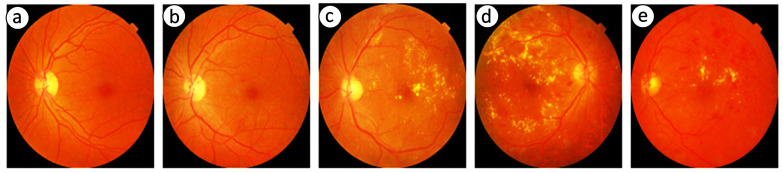
Fundus image showing (**a**) no DR, (**b**) mild, (**c**) moderate, (**d**) severe, and (**e**) PDR.

For better clarity regarding the available fundus images and their categories, Figure 3 illustrates the different stages of DR, ranging from no DR to PDR.

### 3.1. Image Preprocessing

**Figure 4:**
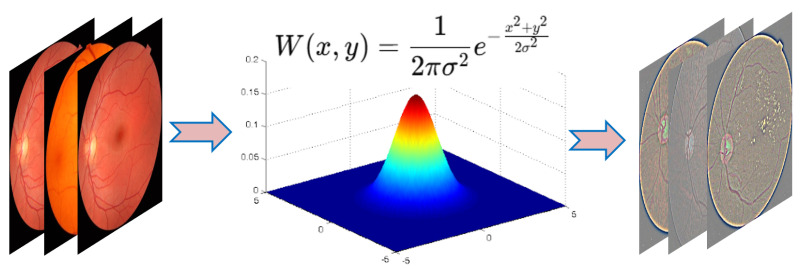
Gaussian filter for noise reduction and smoothing.

Preprocessing is crucial in medical image analysis since it includes many procedures that enhance the quality of the input images [44]. Fundus images obtained from hospitals often contain various abnormalities and artifacts, which present challenges for direct classification using automated methods. Preprocessing aims to improve image quality by mitigating unwanted elements such as noise and enhancing resolution to facilitate more accurate analysis [45]. Common techniques employed include median filtering and histogram equalization, with the choice of the method being contingent upon the image quality and specific elements that need removal [46]. The proposed approach applied essential preprocessing techniques on retinal images acquired from different cameras, which frequently include extraneous black regions. First, the region of interest is cropped from the image to discard unnecessary areas, and then the images are converted to grayscale to simplify the computational process. To maintain uniformity, images were resized to a uniform resolution of 224 × 224 pixels. The primary preprocessing involved noise reduction using Gaussian filtering [47], along with contrast enhancement to improve the clarity of the images while reducing background noise. Gaussian blur is an effective technique for reducing noise, as shown in Figure 4. It may mitigate inconsistencies and flaws without excessive sensitivity to the kind or pattern of noise present in the image. The pivotal element affecting the efficacy of the blur is the selection of the variance value, which dictates the extent of blurring applied to the image. The Gaussian filter calculates a weighted average of adjacent pixel values according to a Gaussian distribution [48].

For an image R(x,y) sized M×N, mathematically, the Gaussian Blur is represented in Equation (1):

(1) $$B_{R}\left(x,y\right)=\sum_{u=-\frac{M}{2}}^{\frac{M}{2}}\sum_{v=-\frac{N}{2}}^{\frac{N}{2}}R\left(u,v\right)\cdot K\left(x-u,\;y-v\right),$$

where

- R(x,y) is the intensity at pixel coordinates (x,y).
- K(u,v) is the Gaussian kernel, defined in Equation (2):

(2) $$K\left(u,v\right)=\frac{1}{2\pi \sigma^{2}}\exp\left(-\frac{u^{2}+v^{2}}{2\sigma^{2}}\right),$$

- u,v are coordinates relative to the kernel center.
- σ denotes the standard deviation that controls the degree of blurring.
- The sum of all kernel weights equals 1, preserving overall brightness.

The smoothed output can be blended with the original image, as shown in Equation (3):

(3) $$R_{\mathrm{final}}\left(x,y\right)=\alpha \;R\left(x,y\right)+\beta \;B_{R}\left(x,y\right)+\gamma ,$$

where α, β, and γ are blending coefficients.

All images are scaled to the [−1,1] interval to ensure consistency in subsequent enhancement stages. Equation (4) expresses this normalization:

(4) $$R_{\mathrm{norm}}\left(x,y\right)=\left(\frac{R(x,y)-\min(R)}{\max(R)-\min(R)}\right)\times 2-1,$$

where

- min(R) and max(R) represent the smallest and largest intensities in the image. This step standardizes images across all datasets, vital for the subsequent enhancement process.
- R(x,y) is the pixel intensity at coordinates (x,y) in the 224×224 grayscale image.
- $R_{\mathrm{norm}}\left(x,y\right)$ is the normalized pixel value in the range [−1,1].

This facilitates consistent and efficient training for the super-resolution models. This preprocessing step ensures that the input images were standardized and clean across the three datasets, which is critical for improving the performance of the subsequent image enhancement phase.

### 3.2. Image Enhancement

In this study, various deep learning models were employed to enhance image resolution, including the Super-Resolution Convolutional Neural Network (SRCNN), Enhanced Deep Super Resolution Network (EDSR), and Swin Transformer for Image Restoration (SwinIR). A novel super-resolution framework, the Hybrid Local–Global Retina Super-Resolution model (HLG-RetinaSR), was developed to improve retinal image quality. Table 1 provides a comparative examination of retinal image enhancement utilizing SRCNN, EDSR, SwinIR, and the proposed HLG-RetinaSR model. The visual outcomes illustrate the efficacy of the models in enhancing image quality.

#### 3.2.1. Super-Resolution Convolutional Neural Network

**Figure 5:**
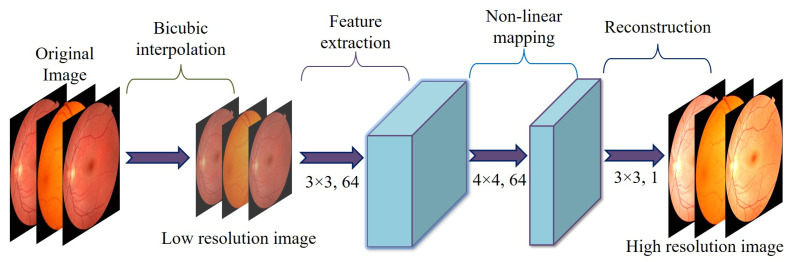
Architecture of SRCNN model for conversion of low-resolution image to high-resolution image.

Super-resolution reconstruction increases the spatial resolution of low-resolution images, similar to other image restoration tasks such as denoising and deblurring. However, while denoising and deblurring replace corrupted pixels without changing the image dimensions, super-resolution generates new pixels to scale up the image. SRCNN, developed by Dong et al. [49] was introduced as the first CNN-based super-resolution model that uses bicubic interpolation to resize input images to the target magnification (e.g., 2×, 3×, or 4×) before applying three convolutional layers. The first layer employs a 9 × 9 kernel to extract features, the second layer uses a 1 × 1 kernel to perform non-linear mapping, and the final 5 × 5 kernel reconstructs the mapped features into a high-resolution output. Figure 5 shows the SRCNN model.

This work uses the input of a single-channel (grayscale) image of size 224×224. The goal is to generate an enhanced image of size 448×448, doubling the original’s width and height. The super-resolution model is based on a three-stage CNN architecture, inspired by the SRCNN approach. The three stages consist of feature extraction, upsampling, and reconstruction, as detailed in the following steps.

- Feature Extraction
  This step applies two convolutional layers to an input image of size 1×224×224. Each layer includes 64 filters, a 3×3 kernel, a stride of 1, and padding of 1. This setup preserves the spatial dimensions and produces feature maps of size 64×224×224.
- Upsampling
  The resolution is expanded from 224×224 to 448×448 using a transposed convolution (deconvolution) layer. This layer is defined with 64 filters, a 4×4 kernel, a stride of 2, and padding of 1, resulting in output feature maps measuring 64×448×448.
- Reconstruction
  The final convolutional layer employs a 3×3 kernel, a stride of 1, and padding of 1. This layer reduces the channel count from 64 down to 1, producing a grayscale output. The resulting super-resolved image has dimensions 1×448×448.

The network is trained using a mean squared error (MSE) loss function, quantifying the disparity between the predicted high-resolution (HR) output image and the target image. The MSE loss is calculated as follows in Equation (5).

(5) $$L\left(\theta \right)=\frac{1}{N}\sum_{k=1}^{N}{∥I_{k}^{HR}-I_{k}^{SR}∥}^{2}$$

where

- *N* is the total number of images in the dataset.
- $I_{k}^{HR}$ represents the ground truth high-resolution image for the *k*-th sample.
- $I_{k}^{SR}$ denotes the super-resolved image predicted by the model.
- θ represents the learnable parameters of the model.

This loss function promotes the network to generate outcomes that maximize the peak signal-to-noise ratio (PSNR), a prevalent measure for assessing the quality of super-resolution images. While the PSNR serves as a valuable metric for quantitative evaluation, it does not consistently correlate with the perceptual quality of retinal images; in some instances, images exhibiting high PSNR may lack visual appeal.

#### 3.2.2. Enhanced Deep Super-Resolution Network

The EDSR model [50] is an improved version of the SRCNN, designed to overcome limitations such as redundant layers and parameters. EDSR removes batch normalization layers and deepens the architecture, making it more effective for high-resolution image reconstruction.

**Figure 6:**
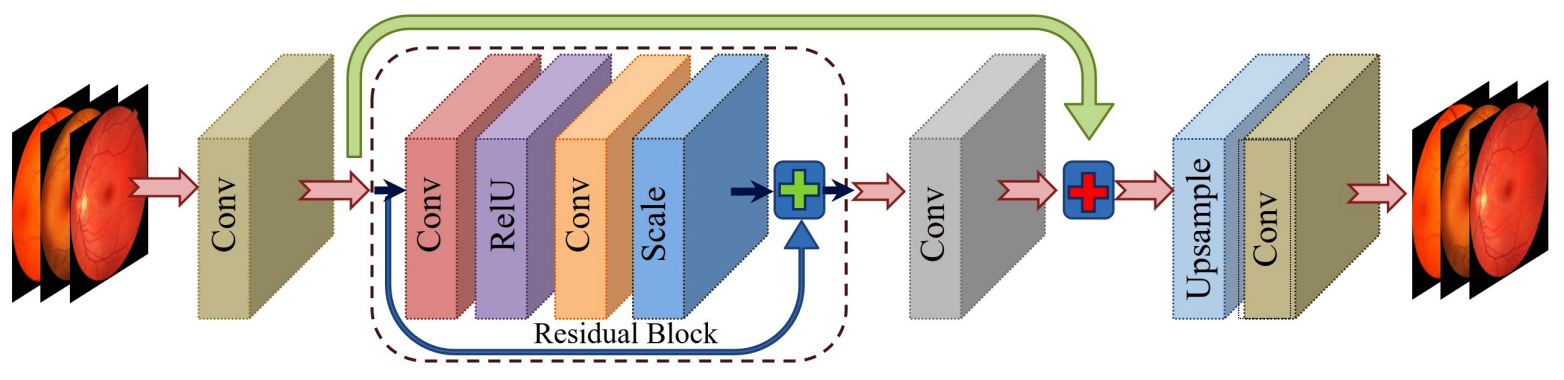
Architecture of EDSR model for conversion of low-resolution image to high-resolution image.

Like SRCNN, MSE loss is used during training to minimize the difference between the predicted super-resolved image and the targeted high-resolution image. Figure 6 shows the EDSR model.

#### 3.2.3. Swin Transformer for Image Restoration

**Figure 7:**
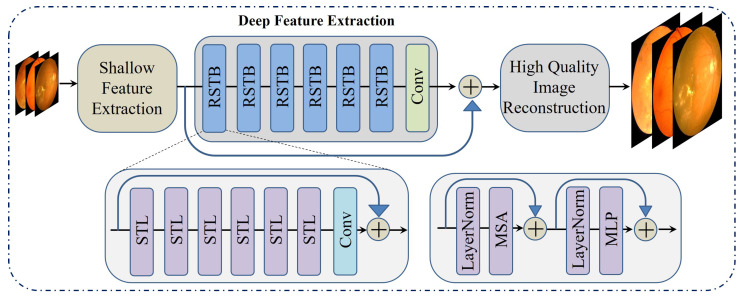
Architecture of SwinIR model for image reconstruction.

SwinIR is built upon the Swin Transformer architecture. It consists of three core components: shallow feature extraction, deep feature extraction, and high-fidelity image reconstruction. Initially, the shallow feature extraction unit utilizes a convolutional layer to gather basic image features, ensuring the preservation of low-frequency details by directly forwarding them to the reconstruction phase. The deep feature extraction unit is built using multiple Residual Swin Transformer Blocks (RSTBs), where each block integrates several Swin Transformer layers to enable localized attention mechanisms and facilitate long-range information exchange. The SwinIR model is shown in Figure 7.

#### 3.2.4. Proposed Novel Hybrid Local–Global Retina Super-Resolution Model

**Figure 8:**
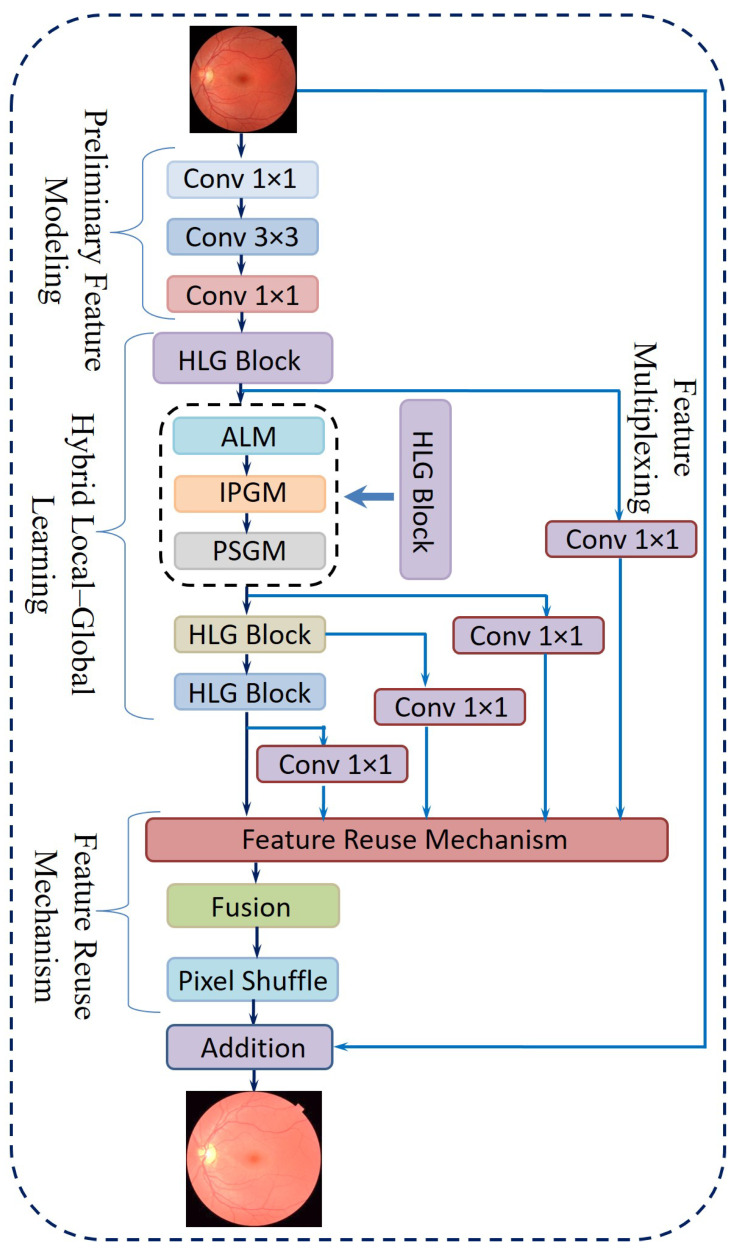
Hybrid Local–Global Retina Super-Resolution (HLG-RetinaSR) block.

**Figure 9:**
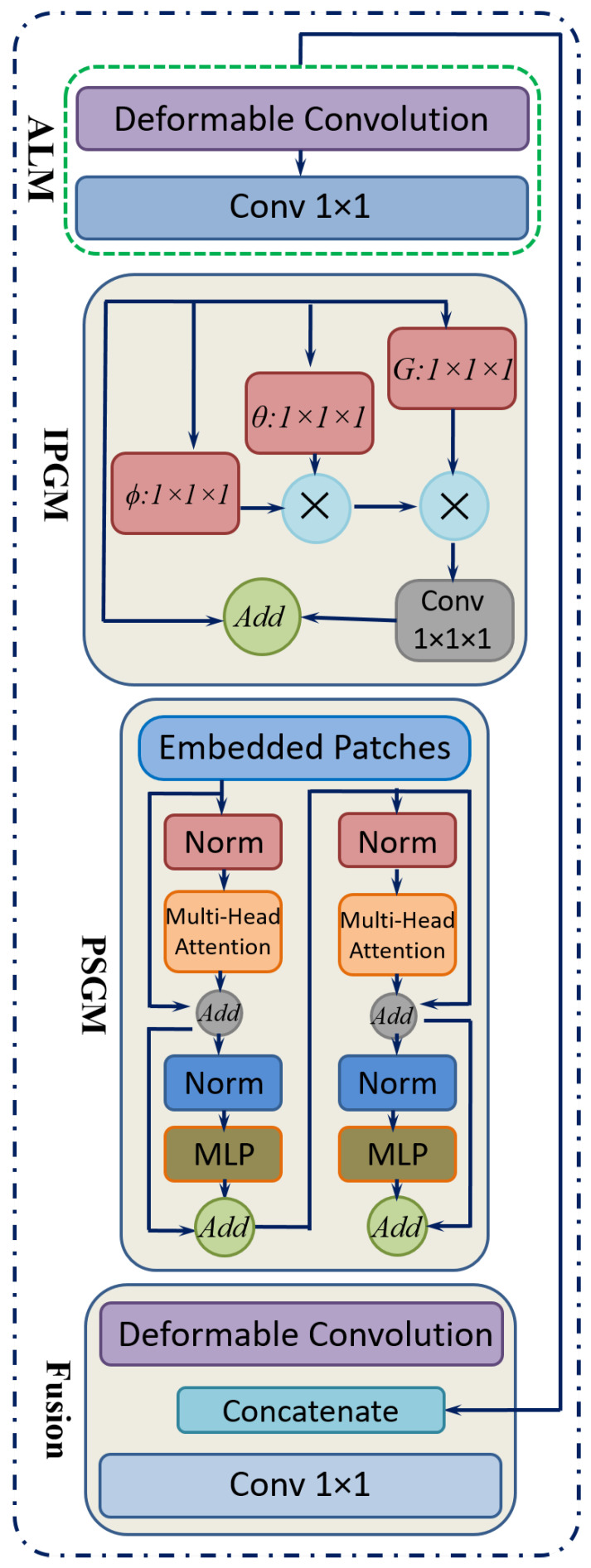
The core block, comprising the HLG blocks of the proposed neural network, includes the ALM, IPGM, and PSGM.

The proposed method is organized into four primary components, namely, Preliminary Feature Modeling, Hybrid Local–Global Learning, the Feature Reuse Mechanism, and Final Reconstruction. Figure 8 shows the architecture of the proposed novel Hybrid Local–Global Retina Super-Resolution Model (HLG-RetinaSR). The following sections provide the details of the operation of each module.

i. Preliminary Feature Modeling
  Conventional convolution-based super-resolution architectures often rely on a single 3 × 3 convolutional layer, which may overlook channel-wise interactions. Drawing inspiration from efficient model design [51], the proposed approach includes the following steps:
  - A point-wise convolution to expand the feature channels.
  - A 3 × 3 convolution to capture spatially distributed features in this augmented space.
  - Another point-wise convolution to merge and reorganize the extracted channels, thereby retaining cross-channel information.
ii. Hybrid Local–Global Learning
  To facilitate localized detail restoration for irregular structures and long-range coherence among possibly distant image regions, a Hybrid Local–Global (HLG) block is introduced. Serving as the core of the proposed neural network, as shown in Figure 9, the HLG block comprises three specialized modules, namely, an Adaptive Local Module (ALM), Inter-Pixel Global Module (IPGM), and Patch-Scope Global Module (PSGM), each described below:
  - ALM: After initial feature modeling, the feature map is passed into the ALM, which is designed to handle spatially irregular or deformed regions common in retinal images. This module leverages a 3×3 deformable convolution [52], which includes learnable offsets and modulations for each kernel location. Unlike fixed-grid convolutions, each position can shift in multiple directions, effectively reshaping the sampling field according to local structures.
    Let *Q* be a set of nine base offsets, for instance, as given in Equation (6):
    (6) Q={(0,0),(0,1),(1,0),(1,1),…,(−1,−1)}.
    For a location *r* in the input, the deformable convolution can be expressed as Equation (7):
    (7) $$y\left(r\right)=\sum_{k=1}^{\left|Q\right|}\alpha_{k}\cdot z\left(r+\delta_{k}\right)\cdot \gamma_{k},$$
    where $\alpha_{k}$ denotes learnable weights, $\delta_{k}$ denotes offset vectors, $\gamma_{k}$ denotes modulation scalars, and *z* denotes the input features. This adaptivity enables the filter’s receptive field to follow structure irregularities more effectively.
    After the deformable layer, a point-wise convolution $P_{\mathrm{wc}}$ further integrates these local features into a refined representation in Equation (8).
    (8) $$H_{\mathrm{ALM}}=P_{\mathrm{wc}}\left(y(r)\right)$$
  - IPGM: In medical diagnosis, comprehending the intricate relationships between distant regions of an organ is crucial, as changes in one area can profoundly affect others. However, traditional convolutional layers, limited by their fixed local receptive fields, often fail to capture these global dependencies effectively. To overcome this constraint, a non-local mechanism [53] is integrated, which establishes correlations between each pixel and all others within the image. This pixel-to-pixel global learning approach is mathematically formulated in Equation (9), where it defines the feature representation at pixel i. The output of the inter-pixel global learning mechanism, denoted as $H_{\mathrm{IPGM}}\left(i\right)$, can be written as
    (9) $$H_{\mathrm{IPGM}}\left(i\right)=\frac{1}{N(x)}\sum_{\forall j}\left[F(x_{i},x_{j})\right]G\left(x_{j}\right),$$
    where F estimates the relationship between position i and position j, and G provides a signal based on those relationships. The normalization term N ensures balanced contributions from all positions. This global pixel–pixel approach contrasts with the ALM’s localized view, effectively restoring large-scale consistencies throughout the image.
  - PSGM: While the IPGM effectively captures pixel-level correlations, it may struggle to identify broader patterns, such as organ boundaries or elongated vessels. To overcome this limitation, the PSGM employs patch-level attention. The feature map is first divided into small patches of 2 × 2 pixels, creating a structured patch sequence. Unlike certain vision transformer-based methods that utilize larger patch sizes of 8 × 8 or 16 × 16 pixels. This approach preserves fine-grained details essential for low-level tasks like super-resolution.
    To further refine feature representation, the PSGM applies a multi-head self-attention mechanism over these patch embeddings, as represented in Equation (10):
    (10) $$H_{\mathrm{PSGM}}=\mathrm{Transformer}\left(X_{\mathrm{patch}}\right),$$
    where $X_{\mathrm{patch}}$ is the restructured patch sequence. This design incorporates two transformer layers with four attention heads, balancing capacity and computational cost. An optional deformable convolution was applied to further refine the patch-oriented features. The resulting feature map was then concatenated with the original input, and a point-wise convolution fused them into the combined output $H_{\mathrm{HLG}}$.
iii. Feature Reuse Mechanism
  Following the HLG block, a feature reuse step was introduced to mitigate redundancy and enhance the utilization of extracted representations. This approach is inspired by prior research [54], which highlights the advantages of revisiting and reusing intermediate features in deep networks. Specifically, the output of each HLG block incorporates a residual skip connection, while a point-wise convolution is applied to compress the dimensionality of $H_{\mathrm{HLG}}$, thereby reducing computational overhead. The final multiplexed features from all HLG blocks are then concatenated and refined through an additional 1 × 1 convolution, leading to the formulation expressed in Equation (11):
  (11) $$M={\mathrm{Conv}}_{1\times 1}\left(\mathrm{Concat}\left[{\mathrm{Conv}}_{1\times 1}\left(H_{\mathrm{HLG}}^{\left(i\right)}\right)\right]\right),$$
  where M denotes the aggregated output prepared for the reconstruction stage.
iv. Final Reconstruction
  The network concludes with a reconstruction phase, which uses
  - A 1 × 1 convolution to further reduce feature dimensions.
  - A Pixel Shuffle layer [55] to upsample the features to the desired resolution.
  - Additive blending of the upsampled output with a simple interpolation of the original input, yielding the final super-resolved image.

### 3.3. Classification of DR

Classification algorithms are fundamental to supervised machine learning, concentrating on the allocation of discrete labels to input data. Using annotated training data, these approaches reveal patterns that may generalize to novel instances. A variety of classifiers exist, each based on distinct theoretical foundations. In the proposed work, the following classifiers were used to evaluate the performance, in terms of accuracy, precision, recall, and F1-score.

#### 3.3.1. Convolution Neural Network

**Figure 10:**
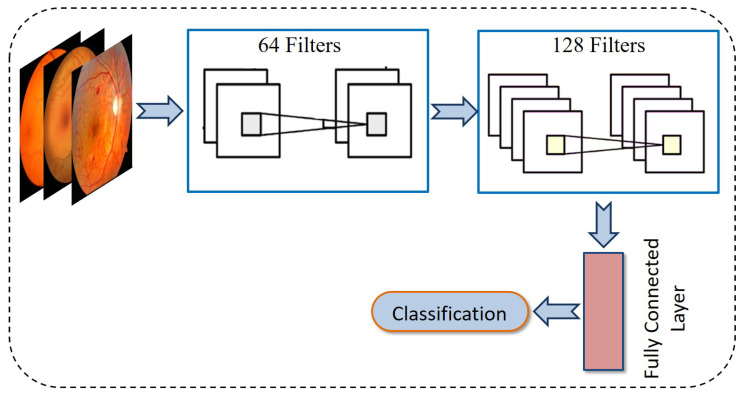
Architecture of CNN model for classification of DR.

A CNN represents a powerful deep learning framework widely utilized for various image processing applications such as object detection, image classification, and image synthesis. As shown in Figure 10, a standard CNN architecture is typically composed of several key components, including convolutional layers, batch normalization, pooling operations, and activation functions. The resulting feature maps are then flattened and fed into a fully connected layer for classification.

#### 3.3.2. DenseNet-121

**Figure 11:**
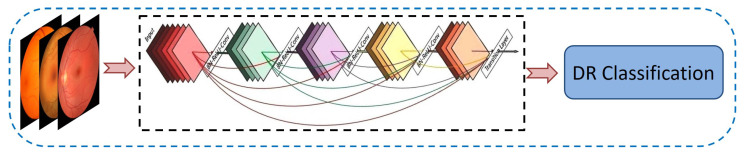
Architecture of DenseNet-121 model for classification of DR.

One prominent example of transfer learning in practice is DenseNet-121 [56], a model pre-trained on large-scale datasets such as ImageNet. Since it already possesses a well-structured hierarchy of learned features, adapting DenseNet-121 for a new task significantly reduces training time and computational requirements, outperforming models trained from scratch. Its densely connected architecture facilitates direct access to gradients and feature maps from all preceding layers, promoting feature reuse and enhancing overall generalization. By fine-tuning its learned feature representations, DenseNet-121 can be seamlessly adapted to meet the specific requirements of a new task. This approach is particularly beneficial for smaller datasets, where training a model from the ground up may be impractical due to data scarcity or computational constraints. Utilizing a pre-trained backbone provides a robust feature extraction phase that can be further optimized for the target problem, making transfer learning an indispensable component of modern deep learning pipelines. Figure 11 illustrates the fundamental structure of the DenseNet-121 model, highlighting its role in efficient feature transfer and adaptation for the classification of DR.

#### 3.3.3. Classification Utilizing RefineNet-U

**Figure 12:**
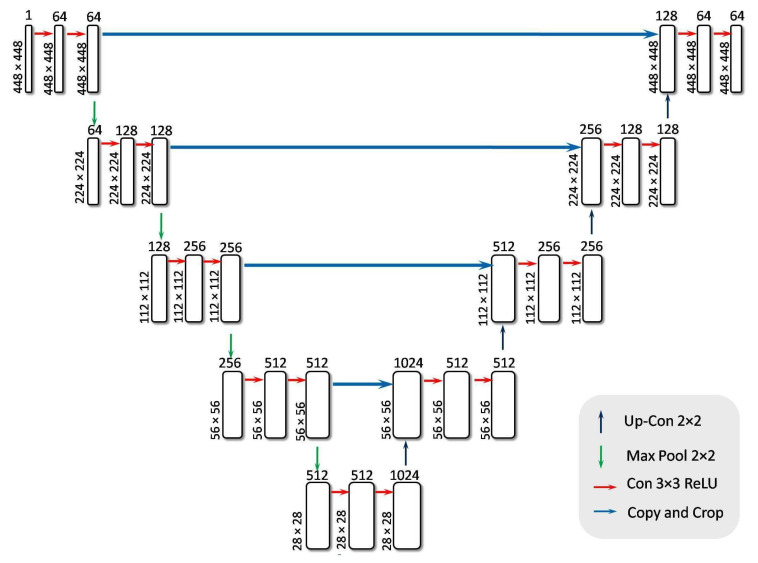
Architecture of RefineNet-U model.

Ronneberger et al. [57] introduced U-Net, a convolutional neural network primarily designed for medical image segmentation. Its distinctive U-shaped architecture consists of a contracting (encoder) path and an expanding (decoder) path, connected by a bridging layer. In the encoder, each stage reduces spatial resolution through convolution and pooling operations, distilling essential features. The decoder then uses transposed convolutions to progressively restore spatial dimensions while fusing information from corresponding encoder layers, preserving both local detail and broader contextual information. In the proposed approach, a modified U-Net model named RefineNet-U was developed, which incorporates an additional dense classification layer to enable robust disease grading. The architecture of RefineNet-U is shown in Figure 12. The model consists of three main components: an encoder block, a bridge block, and a decoder block. The encoder extracts and condenses feature maps, the bridge connects the encoder and decoder at the bottleneck, and the decoder reconstructs spatial structure before passing the refined features to a fully connected layer for final classification. This architecture preserves U-Net’s strength in extracting spatially precise features, which is particularly valuable for highlighting disease-relevant regions, while also delivering strong performance in multiclass classification tasks. The functions and specifics of each component are discussed in the following sections:

- Encoder Block
  The encoder block is a feature extractor that encodes input images through multiple sequential blocks. Each encoder block contains two convolutional layers, both activated by the ReLU function, followed by a max pooling layer. ReLU effectively nullifies any negative input by assigning it a zero slope. While the convolutional layers increase the depth (number of feature channels), the max pooling layer halves the spatial dimensions of the feature maps without reducing their depth.
  In this study, retinal images of size 448 × 448 × 1 were employed as input. The first convolutional layer, consisting of 64 filters, transforms the input image into a feature map of dimensions 448 × 448 × 64. The max-pooling operation, depicted by a downward arrow in Figure 12, is then applied to reduce the feature map size to 224 × 224 × 64. The network architecture incorporates four encoder blocks, where each block (except the first) receives the output feature map from its preceding block. The output of the final encoder block is passed to a bridge block for further processing. The convolution and downsampling procedures mentioned above are formally described in Equations (12) and (13).
  (12) $$f\left(x\right)=\left\{\begin{array}{cc} 0, & \mathrm{if}\;x<0\\ x, & \mathrm{if}\;x\geq 0 \end{array}\right.$$
  (13) Conv(i)=activation(convolution(input(i),filter(i),kernel_size(i)))
  This process effectively extracts and compresses important features, preparing the data for classification into 5 classes.
- Bridge Block
  The bridge block ensures a seamless flow of encoded features from the encoder to the decoder. Table 2 outlines the specifics of this component, which contains two convolutional layers using ReLU for activation. After the four encoder blocks have downsampled the 448 × 448 × 1 input to 28 × 28 × 512, the bridge block transforms these feature maps to 28 × 28 × 1024. The resulting output is a high-level representation of the retinal images, which is then passed on to the decoder network for further processing. It is described in Equation (14).
  (14) $$\begin{array}{cc} \mathrm{Bottleneck}= & \mathrm{activation}(\mathrm{convolution}(\mathrm{downsampled},\\ \; & \mathrm{filter\_bottleneck},\;\mathrm{kernel\_size\_bottleneck})) \end{array}$$
  This bottleneck layer creates an abstract representation of the input image, capturing the essential features required for classification. This compressed feature map is then forwarded to the decoder block for further processing.
- Decoder Block
  The decoder network reconstructs the final segmentation mask after the bridge block delivers its high-level representation.
  The decoder comprises decoder blocks, each containing a 2D transposed convolution for upsampling, a concatenation step that merges data from the corresponding encoder level, and two standard 2D convolutions activated by ReLU.
  In Figure 12, the upward-pointing orange arrow denotes the transpose convolution process that enlarges the spatial dimensions.
  Specifically, the feature map is scaled up from 28 × 28 × 1024 to 56 × 56 × 512, and then concatenated with the matching encoder output (also 56 × 56 × 512) to form a combined feature map of 56 × 56 × 1024. By integrating earlier-layer features in this way, the decoder can refine its predictions for more accurate segmentation. Eventually, after repeating the upsampling process through all decoder blocks, the network restores the spatial resolution to the original input size of 448 × 448 × 1, as shown in Equation (15). This final multi-channel feature map is treated as the UNet output and is then passed to flattened and dense layers for subsequent classification tasks.
  (15) $$\mathrm{concatenated\_image}=\mathrm{concatenate}\left(\mathrm{upsampled\_image},\mathrm{encoder\_image}\right)$$
  This process ensures that information is preserved and utilized effectively for accurate classification, making the decoder block a critical component in the classification task.
- Flattening and Dense Layer
  The RefineNet-U generates a multi-dimensional feature map through its convolutional layer, which is then transformed into a one-dimensional vector representation. To prevent the risk of overfitting, a dropout mechanism with a probability of 0.3 is applied. Following this, the resulting vector undergoes processing through a sequence of two fully connected (dense) layers. In these dense layers, every neuron establishes a direct link with all neurons from the preceding layer, ensuring complete interconnection. Additionally, the Leaky Rectified Linear Unit (LeakyReLU) activation function is employed within the dense layers to introduce non-linearity and enhance learning performance. In contrast to conventional ReLU, LeakyReLU retains negative values by assigning them a minor slope, as specified in Equation (16). The network culminates in a dense output layer that employs the softmax activation function. The softmax function computes the probabilities for each class, yielding a probability distribution in which the total of all probabilities equals one. The class with the greatest likelihood is deemed the projected class. The softmax function is shown in Equation (17). Subsequent to the network’s construction, the model undergoes training on the dataset and is then evaluated for classification accuracy using the test data. Table 3 shows the mathematical model of RefineNet-U. Table 4 presents a summary of the parameter values used in the encoder, bridge, decoder, flattening, and dense layers.
  (16) $$f\left(x\right)=\left\{\begin{array}{cc} x, & \mathrm{if}\;x\geq 0\\ a\cdot x, & \mathrm{if}\;x<0 \end{array}\right.$$
  where *x* is the input value and a is a small constant for the negative slope in the LeakyReLU activation function.
  (17) $${\left(y\right)}_{i}=\frac{\exp(y_{i})}{\sum_{j=1}^{n}\exp\left(y_{j}\right)}$$
  where *y* is the input vector, ${\left(y\right)}_{i}$ is the *i*-th element of the input vector, and *n* is the total number of classes.

**Table 2:** Architecture description.

Block Name	Layer Type	Count of Layers	Parameter	Value
Encoder Block	2D Convolution Layer	2 per block (4 blocks)	Filters	64, 128, 256, 512
			Kernel size	3 × 3
			Activation	ReLU
	Max Pooling layer	1 per block (4 blocks)	Pool size	2 × 2
			stride	2
	Dropout Layer	1 (after the last block)	Dropout Rate	0.5
Bridge Block	2D Convolution Layer	2	Filters	1024
			Kernel Size	3 × 3
			Activation	ReLU
			Padding	same
Decoder Block	Transposed 2D Convolution layer	1 per block (4 blocks)	Filters	512, 256, 128, 64
			Kernel size	2 × 2
			Activation	ReLU
	2D Convolution Layer	2 per block (4 blocks)	Filters	512, 256, 128, 64
			Kernel size	3 × 3
			Activation	ReLU
	Concatenation layer	1 per block (4 blocks)	-	N/A
Flattening Layer	Flattening Layer	1	-	N/A
Dense Block	Dense Layer	2	Neurons	128, 5
			Activation (Dense 1)	ReLU
			Activaton (Dense 2)	Softmax
General	Batch Normalization Layer	Applied after Convolution Layers	-	N/A

**Table 3:** Mathematical formulation of RefineNet-U architecture.

Stage	Equation	Notations
Input	$x^{\left(0\right)}\in R^{H\times W\times C}$	$x^{\left(0\right)}$: input fundus image with height *H*, width *W*, and channels *C*; R: the set of real numbers
Encoder	$\begin{array}{cc} u^{\left(l,1\right)} & =\sigma (W^{\left(l,1\right)}*x^{\left(l-1\right)}+b^{\left(l,1\right)})\\ u^{\left(l,2\right)} & =\sigma (W^{\left(l,2\right)}*u^{\left(l,1\right)}+b^{\left(l,2\right)})\\ x^{\left(l\right)} & =u^{\left(l,2\right)} \end{array}$	$u^{\left(l,1\right)}$,$u^{\left(l,2\right)}$: intermediate activations at level *l*; $x^{\left(l\right)}$: encoder feature map; σ: ReLU activation; ∗: convolution operation; $W^{\left(l\right)},\;b^{\left(l\right)}$: weights and biases
Pooling	$x_{\mathrm{pool}}^{\left(l\right)}=\mathrm{MaxPool}\left(x^{\left(l\right)}\right)$	$x_{\mathrm{pool}}^{\left(l\right)}$: reduces spatial resolution of $x^{\left(l\right)}$
Bottleneck	$\begin{array}{cc} b & =\sigma (W^{\left(L+1,1\right)}*x_{\mathrm{pool}}^{\left(L\right)}+b^{\left(L+1,1\right)})\\ b & =\sigma (W^{\left(L+1,2\right)}*b+b^{\left(L+1,2\right)}) \end{array}$	*b*: bottleneck feature representation; L: last encoder layer index
Decoder	${\tilde{u}}^{\left(l\right)}=\mathrm{Up}\left({\tilde{x}}^{\left(l+1\right)}\right)$	Upsampling: Up enlarges spatial resolution of decoder feature map ${\tilde{x}}^{\left(l+1\right)}$ to ${\tilde{u}}^{\left(l\right)}$
	${\tilde{c}}^{\left(l\right)}=\mathrm{Cat}\left({\tilde{u}}^{\left(l\right)},x^{\left(l\right)}\right)$	Skip connection: Cat(·) concatenates decoder upsampled features ${\tilde{u}}^{\left(l\right)}$ with $x^{\left(l\right)}$; result: ${\tilde{c}}^{\left(l\right)}$
	$\begin{array}{cc} {\tilde{v}}^{\left(l,1\right)} & =\sigma ({\tilde{W}}^{\left(l,1\right)}*{\tilde{c}}^{\left(l\right)}+{\tilde{b}}^{\left(l,1\right)})\\ {\tilde{v}}^{\left(l,2\right)} & =\sigma ({\tilde{W}}^{\left(l,2\right)}*{\tilde{v}}^{\left(l,1\right)}+{\tilde{b}}^{\left(l,2\right)})\\ {\tilde{x}}^{\left(l\right)} & ={\tilde{v}}^{\left(l,2\right)} \end{array}$	Two convolutions + ReLU, ${\tilde{v}}^{\left(l,1\right)}$; ${\tilde{v}}^{\left(l,2\right)}$: intermediate activations; ${\tilde{x}}^{\left(l\right)}$: refined decoder feature map
Output	${\hat{y}}_{ijc}=\frac{\exp(\ell_{ijc})}{\sum_{k=1}^{C_{\mathrm{out}}}\exp\left(\ell_{ijk}\right)},\;\ell_{ij}=W_{\mathrm{out}}*{\tilde{x}}_{ij}^{\left(1\right)}+b_{\mathrm{out}}$	Final output: logits $\ell_{ij}$ at pixel (i,j); $W_{\mathrm{out}},b_{\mathrm{out}}$: weights and biases; ${\hat{y}}_{ijc}$: probability of class *c* at pixel (i,j); $C_{\mathrm{out}}=5$ (DR severity grades)
Prediction	${\hat{Y}}_{ij}=\arg\max_{c}{\hat{y}}_{ijc}$	Pixel (i,j) assigned to most probable class ${\hat{Y}}_{ij}$ from predicted probabilities ${\hat{y}}_{ijc}$
Loss Function	$L_{\mathrm{CE}}=-\sum_{i,j}\sum_{c=1}^{C_{\mathrm{out}}}y_{ijc}\log\left({\hat{y}}_{ijc}\right)$	Categorical cross-entropy: compares predicted ${\hat{y}}_{ijc}$ with ground truth one-hot label $y_{ijc}$ at each pixel (i,j); $\tau =L_{CE}$ is total training loss

**Table 4:** Performance metrics and image quality measures.

Parameter Name	Definition	Formula
Accuracy (Acc)	Measures the proportion of correctly predicted cases among all cases.	$\mathrm{Acc}=\frac{T_{P}+T_{N}}{T_{P}+T_{N}+F_{P}+F_{N}}$
Precision (Prc)	Proportion of correctly predicted DR cases among all predicted DR cases.	$\mathrm{Prc}=\frac{T_{P}}{T_{P}+F_{P}}$
F1-Score (F1)	Harmonic mean of precision and recall, balancing both metrics.	$F_{1}=\frac{2\times \mathrm{Prc}\times \mathrm{Rcl}}{\mathrm{Prc}+\mathrm{Rcl}}$
Recall (Rcl)	Proportion of accurately predicted DR cases among actual DR cases.	$\mathrm{Rcl}=\frac{T_{P}}{T_{P}+F_{N}}$
Specificity (Spc)	Measures the model’s ability to correctly classify non-DR cases.	$\mathrm{Spc}=\frac{T_{N}}{T_{N}+F_{P}}$
Mean Squared Error (MSE)	Measures the average squared difference between original and processed image intensities.	$\mathrm{MSE}=\frac{1}{mn}\sum_{i=0}^{m-1}\sum_{j=0}^{n-1}{\left[I\left(i,j\right)-K\left(i,j\right)\right]}^{2}$
Peak Signal-to-Noise Ratio (PSNR)	Evaluate the ratio of signal power to noise interference in an image.	$\mathrm{PSNR}=10\log_{10}\left(\frac{MAX_{I}^{2}}{\mathrm{MSE}}\right)$
Structural Similarity Index Measure (SSIM)	Assess image similarity based on structure, brightness, and contrast.	$\mathrm{SSIM}\left(x,y\right)=\frac{\left(2\mu_{x}\mu_{y}+C_{1}\right)\left(2\sigma_{xy}+C_{2}\right)}{\left(\mu_{x}^{2}+\mu_{y}^{2}+C_{1}\right)\left(\sigma_{x}^{2}+\sigma_{y}^{2}+C_{2}\right)}$

The softmax activation function transforms an input vector into a probability distribution over n classes, as shown in Equation (16). It is commonly used for multiclass classification, particularly when the model needs to distinguish among more than two categories. In this context, softmax helps categorize images into multiple classes by assigning a probabilistic interpretation to the model’s outputs. Specifically, it produces a probability value for each class, ensuring all such values sum to 1.

This probabilistic approach is advantageous for decision-making because it provides a measure of the model’s confidence for each class. Instead of returning a single, definitive label, softmax outputs continuous values between 0 and 1, indicating how likely it is that the input belongs to each category. This detailed distribution is crucial for multiclass tasks, as it offers more nuanced insights into the classification outcome and assists in thresholding decisions. Moreover, softmax can be seamlessly integrated into existing model architectures, enabling the handling of multiple classes without substantial modifications.

After enhancing, the images were analyzed using a specialized architecture called RefineNet-U, an advanced adaptation of the U-Net model designed for precise categorization of retinal images into five classes: no DR, mild DR, moderate DR, severe DR, and proliferate DR. This architecture combines a contracting pathway for multi-scale feature extraction with an expanding pathway that ensures accurate localization of abnormalities. The skip connections between corresponding layers in the contracting and expanding pathways preserve essential spatial information, significantly enhancing the model’s capability to accurately determine the severity of DR.

## 4. Results and Discussion

The classifiers CNN, DenseNet121, and RefineNet-U were evaluated based on accuracy, precision, recall, F1-score, specificity, sensitivity, training and testing accuracy, and training and testing loss to assess their effectiveness in DR prediction.

### 4.1. Experimental Setup

The experimental configuration for DR classification involved a laptop with 16 GB of RAM, an Intel Core i7 CPU, and an NVIDIA RTX 2080 GPU to facilitate the fast training of the deep learning models. The machine operated on a 64-bit Windows OS. The tests were conducted using Python 3.8 as the programming environment, using TensorFlow/Keras for the construction and training of deep learning models.

Table 4 shows the performance metrics utilized to measure the efficiency of DR classification and the measures used to calculate image quality.

### 4.2. Data Acquisition

To assess the effectiveness of the proposed approach, three widely recognized fundus image datasets were utilized. Each of these datasets introduces distinct challenges, such as the presence of minute lesions, variations between different classes, and imbalanced class representation. A detailed overview of the selected datasets used for evaluation is provided below. The details of each dataset are also provided in Table 5.

#### 4.2.1. APTOS-2019 Dataset (Dataset I)

The first dataset utilized in this study was sourced from the publicly available Asia Pacific Teleophthalmology Society (APTOS 2019) challenge [58]. This dataset consists of a total of 3662 annotated retinal fundus images along with 1928 unannotated images. For this research, only the labeled set of 3662 images was considered. These images are categorized into five specific classes, each indicating a different stage of diabetic retinopathy (DR), ranging from Class 0 (no DR or healthy), Class 1 (mild DR), Class 2 (moderate DR), Class 3 (severe DR), and Class 4 (PDR).

#### 4.2.2. Messidor-2 Dataset (Dataset II)

The second dataset is the Messidor-2 dataset, consisting of 1800 RGB retinal images, each accompanied by an Excel file containing medical observations [59]. These images are stored with an 8-bit color depth of resolutions 1440 × 960, 2240 × 1488, or 2304 × 1536 pixels. Out of the 1800 images, 1200 were captured with dilated pupils, while 600 were taken without dilation. This dataset is also publicly available.

#### 4.2.3. DDR Dataset (Dataset III)

The third dataset utilized is the DDR (Diabetic Retinopathy) dataset, which comprises a total of 13,673 color fundus images and is publicly accessible via Kaggle [60]. These retinal images were collected from 9598 individuals, whose ages span from 1 to 100 years, with the mean age being approximately 54.13 years. Of the total number of images, 48.23% correspond to male patients, while 51.77% belong to female patients. This particular dataset was sourced from a repository available on GitHub.

Across all three datasets used in this study, APTOS 2019, Messidor-2, and DDR, the grading of DR is consistent with the International Clinical Diabetic Retinopathy Disease Severity Scale (ICDR-DSS), ensuring alignment with established clinical practice. The five stages are universally defined by pathological findings: no DR (absence of lesions), mild NPDR (microaneurysms only), moderate NPDR (microaneurysms with hemorrhages, exudates, or mild vascular changes), severe NPDR (following the “4-2-1 rule” with extensive hemorrhages, venous beading, or IRMA), and PDR (presence of neovascularization with or without vitreous or preretinal hemorrhage). While APTOS 2019 emphasizes ophthalmologist-annotated images from diverse screening populations, Messidor-2 provides a benchmark dataset with high-quality expert labels, and DDR contributes large-scale annotated images with lesion-specific consistency. The adoption of these datasets in this study ensures that automated grading is grounded in clinically recognized criteria rather than arbitrary labels, thereby enhancing both interpretability and medical relevance of the proposed framework.

### 4.3. Experimental Results

#### 4.3.1. Accuracy and Loss Using Dataset I

**Figure 13:**
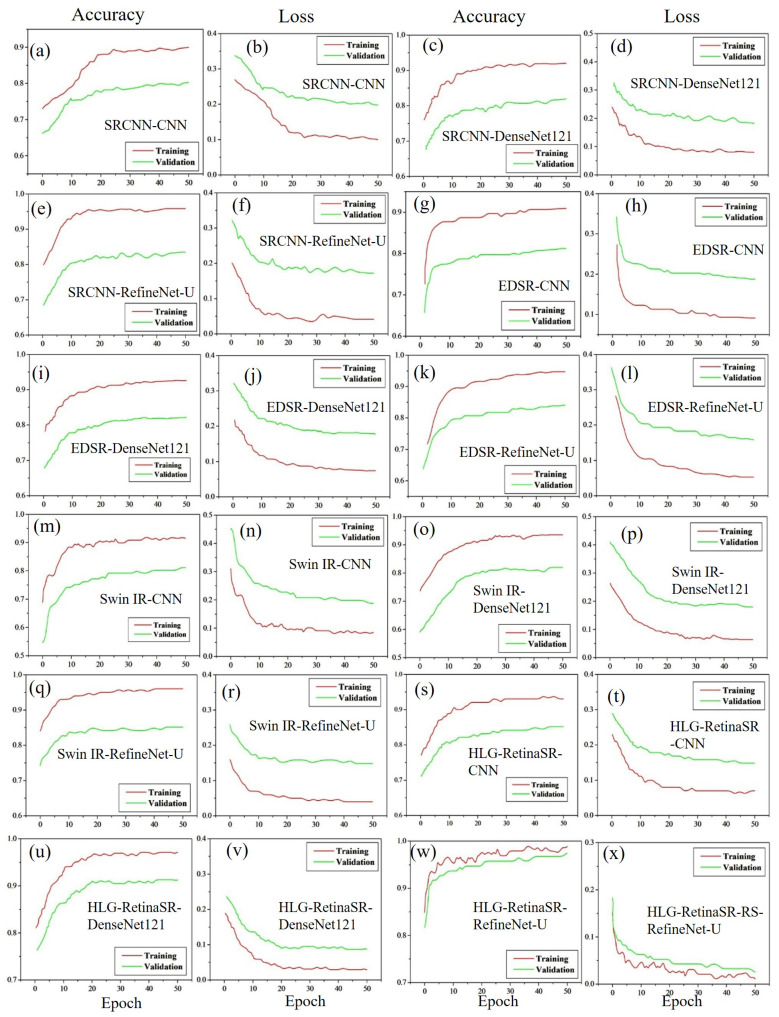
Accuracy and loss graphs for Dataset I.

Figure 13 presents the accuracy and loss graphs for Dataset I, illustrating the performance of various classification models in conjunction with different image enhancement techniques. These models are utilized to enhance and classify the various stages of DR. The classification outcomes differ across models when SRCNN is applied for image enhancement. The CNN model, when integrated with SRCNN, achieves an accuracy of 80.21% (Figure 13a) while maintaining a corresponding loss of 19.09% (Figure 13b). A noticeable improvement is observed with DenseNet-121, which increases accuracy to 81.07% (Figure 13c) while reducing the loss to 17.06% (Figure 13d). The highest performance within this category is attained with RefineNet-U, yielding an accuracy of 83.47% (Figure 13e) and a significantly lower loss of 15.83% (Figure 13f).

Further improvement in accuracy is observed when employing EDSR. The CNN model integrated with EDSR records the lowest accuracy in this category at 81.21% (Figure 13g) with the highest loss of 18.09% (Figure 13h). The accuracy improves with DenseNet-121, reaching 82.15% (Figure 13i) while the loss decreases to 17.15% (Figure 13j). The best results within the EDSR-enhanced models are obtained with RefineNet-U, achieving an accuracy of 84.01% (Figure 13k) and a loss of 15.2% (Figure 13l). A similar trend is observed with SwinIR as the enhancement technique. The combination of SwinIR with CNN results in an accuracy of 81.16% (Figure 13m) with a loss of 18.14% (Figure 13n). A slight improvement is noted when SwinIR is used with DenseNet-121, increasing the accuracy to 81.97% (Figure 13o) and reducing the loss to 17.33% (Figure 13p). The highest performance in this category is achieved with RefineNet-U, which attains an accuracy of 85.16% (Figure 13q) and the lowest loss of 14.14% (Figure 13r).

Among all enhancement techniques, the best classification performance is obtained with HLG-RetinaSR. The accuracy consistently increases from CNN to DenseNet-121 and reaches its peak with RefineNet-U. Specifically, when HLG-RetinaSR is combined with CNN, an accuracy of 82.09% (Figure 13s) is achieved with a loss of 16.04% (Figure 13t). The accuracy is further improved with DenseNet-121, reaching 91.32% (Figure 13u) with a significantly lower loss of 7.98% (Figure 13v). The most optimal combination is achieved with RefineNet-U, which delivers an impressive accuracy of 97.42% (Figure 13w) and a minimal loss of 1.88% (Figure 13x). These results conclusively demonstrate that HLG-RetinaSR is the most effective image enhancement technique, yielding the highest classification accuracy when combined with RefineNet-U. Table 6 shows the performance evaluation for Dataset I.

#### 4.3.2. Accuracy and Loss Using Dataset II

**Figure 14:**
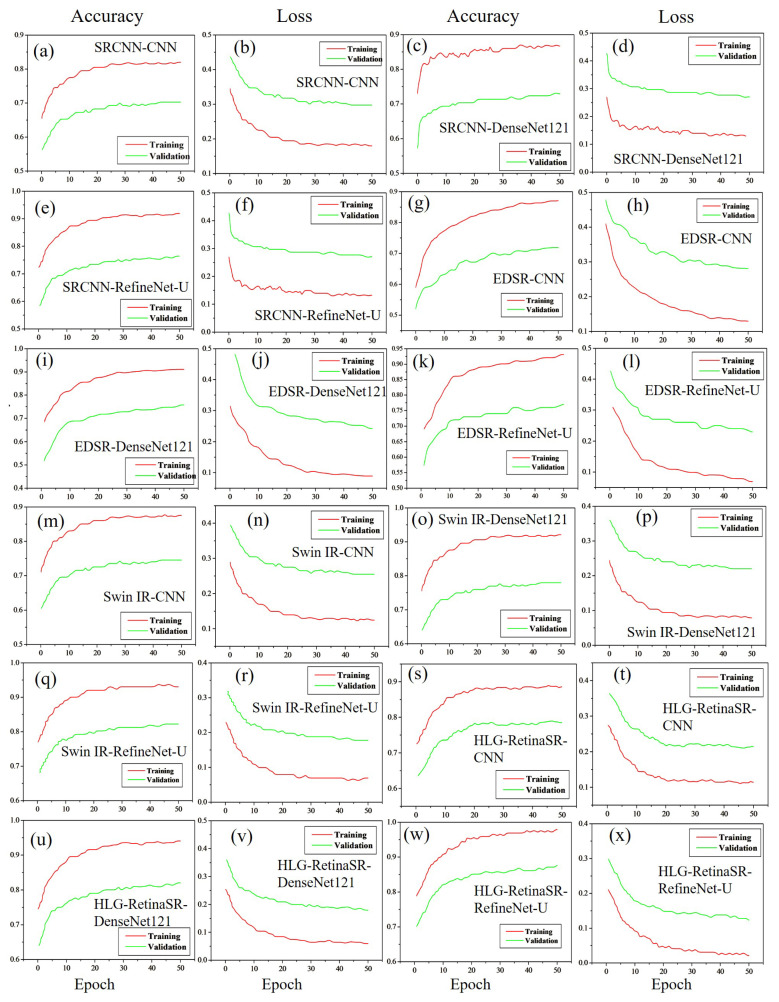
Accuracy and loss graphs for Dataset II.

Figure 14 presents the accuracy and loss trends for Dataset II. This evaluation examines how different enhancement methods influence classification performance. Among them, SRCNN demonstrates varying accuracy and loss rates when paired with different classification models. As shown in Figure 14a, the combination of SRCNN with CNN achieves an accuracy of approximately 70.00%, accompanied by a loss of 29.03% (Figure 14b). A slight improvement is observed with DenseNet-121, increasing the accuracy to 73.00% (Figure 14c) while reducing the loss to 29.97% (Figure 14d). The highest performance within this enhancement category is achieved with RefineNet-U, which attains an accuracy of nearly 76.00% (Figure 14e) and a significantly lower loss of 22.89% (Figure 14f).

Further enhancement is observed when EDSR is applied in conjunction with classification models. The CNN-EDSR combination yields the lowest accuracy of 71.87% (Figure 14g) and the highest loss of 27.43% (Figure 14h) compared to other models. However, performance improves with DenseNet-121, reaching an accuracy of 75.77% with a reduced loss of 24.23% (Figure 14i,j). The highest accuracy in this category is achieved when EDSR is paired with RefineNet-U, attaining 76.99% accuracy (Figure 14k) and a loss of 23.01% (Figure 14l). A similar trend is observed with SwinIR, where accuracy gradually improves across classification models. When combined with CNN, SwinIR achieves an accuracy of 74.53% (Figure 14m) with a loss of 24.47% (Figure 14n). The performance is slightly enhanced with DenseNet-121, yielding an accuracy of 77.98% (Figure 14o) and a reduced loss of 21.02% (Figure 14p).

The best results within this category are obtained when SwinIR is integrated with RefineNet-U, achieving an accuracy of 82.22% (Figure 14q) with a minimal loss of 17.78% (Figure 14r). The most significant improvement is observed with the HLG-RetinaSR enhancement method, which outperforms all other techniques. The accuracy follows an increasing trend from CNN to DenseNet-121 and reaches its peak with RefineNet-U. When combined with CNN, HLG-RetinaSR achieves an accuracy of 79.09% (Figure 14s) with a loss of 20.21% (Figure 14t). A further enhancement is noted with DenseNet-121, which achieves 82.03% accuracy (Figure 14u) with a significantly reduced loss of 11.27% (Figure 14v). The best classification performance is recorded when HLG-RetinaSR is paired with RefineNet-U, yielding an exceptional accuracy of 87.66% (Figure 14w) and a loss of 12.34% (Figure 14x). The higher loss observed in this dataset is primarily due to the smaller number of images compared to the other datasets. However, the results also clearly indicate that HLG-RetinaSR is the most effective image enhancement technique. When it is combined with RefineNet-U, it delivers superior classification accuracy, making this integration an auspicious approach for enhancing diabetic retinopathy detection through advanced image enhancement. Table 7 depicts the performance evaluation for Dataset II.

#### 4.3.3. Accuracy and Loss Using Dataset III

**Figure 15:**
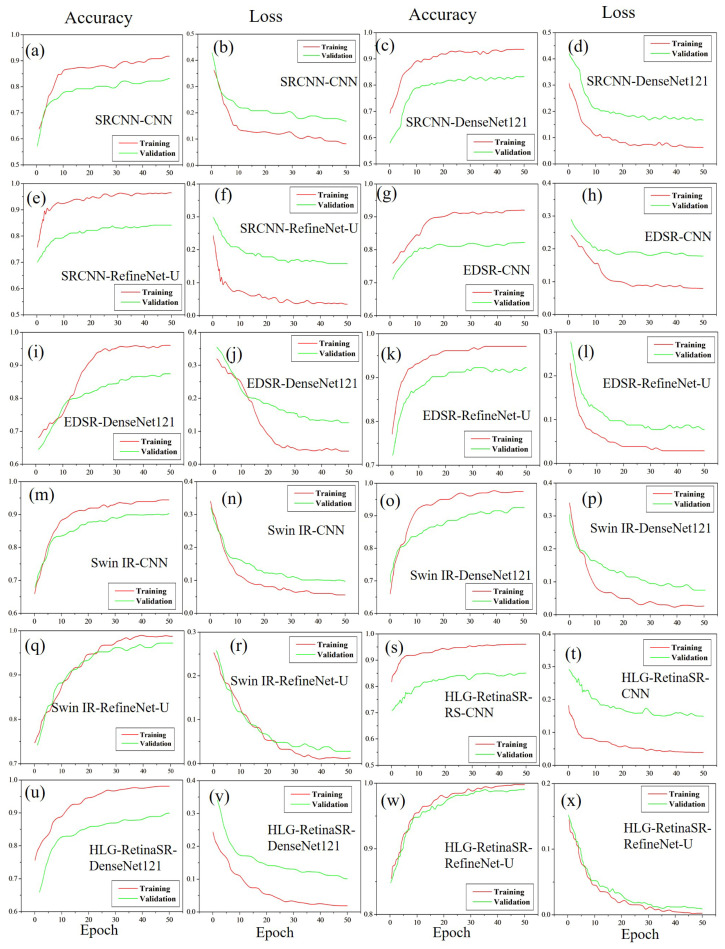
Accuracy and loss graphs for Dataset III.

Figure 15 presents the accuracy and loss graphs for Dataset III, showcasing the impact of different image enhancement techniques on the classification of DR across various models. The effectiveness of SRCNN varies depending on the classification model used. When paired with CNN, it achieves an accuracy of 83.17% (Figure 15a) with a corresponding loss of 15.83% (Figure 15b). Marginal improvement is observed with DenseNet-121, where accuracy rises to 83.66% (Figure 15c) and loss reduces to 15.34% (Figure 15d). The highest accuracy with SRCNN is attained using RefineNet-U, reaching 84.14% (Figure 15e) with a lower loss of 14.86% (Figure 15f).

Further performance enhancement is achieved with EDSR, though its effectiveness varies across models. Among the tested models, CNN with EDSR demonstrates the lowest accuracy at 82.18% (Figure 15g) and the highest loss of 16.82% (Figure 15h). However, integrating EDSR with DenseNet-121 significantly improves accuracy to 87.4% while reducing the loss to 11.6% (Figure 15i,j). The best performance within the EDSR framework is observed with RefineNet-U, which achieves an accuracy of 92.23% (Figure 15k) and minimizes loss to 6.77% (Figure 15l). SwinIR follows a similar trend, demonstrating progressive accuracy gains across models. When combined with CNN, it achieves 90.29% accuracy (Figure 15m) with a loss of 8.71% (Figure 15n). A moderate improvement is noted with DenseNet-121, where accuracy increases to 92.54% (Figure 15o) while loss drops to 6.46% (Figure 15p). The highest accuracy with SwinIR is attained using RefineNet-U, reaching an impressive 97.02% (Figure 15q) with a minimal loss of 2.43% (Figure 15r).

The most effective enhancement–classification combination is achieved with HLG-RetinaSR, consistently improving classification accuracy. When applied to CNN, HLG-RetinaSR achieves 85.11% accuracy (Figure 15s) with a loss of 13.89% (Figure 15t). Its integration with DenseNet-121 further elevates accuracy to 89.9% while reducing loss to 9.1% (Figure 15u,v). The highest classification performance is obtained when HLG-RetinaSR is paired with RefineNet-U, achieving an outstanding accuracy of 99.11% (Figure 15w) with an exceptionally low loss of 0.78% (Figure 15x). These findings establish HLG-RetinaSR as the most effective image enhancement technique, delivering superior classification accuracy when combined with RefineNet-U. The accuracy consistently improves from CNN to DenseNet-121 and reaches its peak with RefineNet-U, reaffirming HLG-RetinaSR as the optimal enhancement model for DR classification. The performance evaluation for Dataset III is presented in Table 8.

### 4.4. Classification Analysis

**Figure 16:**
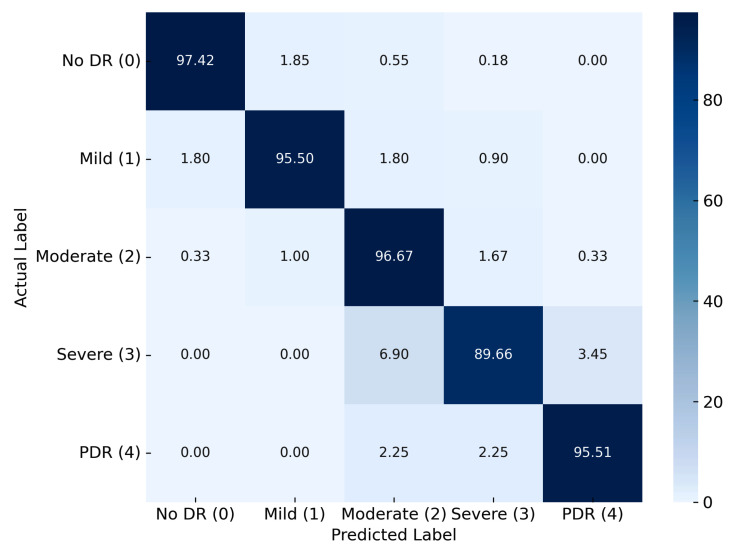
Confusion matrix for Dataset I.

The performance of the proposed DR classification framework was further evaluated using normalized confusion matrices across three distinct datasets, I, II, and III, each encompassing five DR severity levels: no DR (0), mild DR (1), moderate DR (2), severe DR (3), and PDR (4). For Dataset I, the confusion matrix demonstrates the strong classification capability of the model, which combines HLG-RetinaSR preprocessing with the RefineNet-U architecture. High classification accuracies were achieved for no DR (97.42%), moderate DR (96.67%), and PDR (95.51%), while mild DR also exhibited strong performance with 95.50% accuracy, as shown in Figure 16. Although the accuracy for severe DR was slightly lower at 89.66%, overall misclassification remained minimal, indicating the model’s robust ability to differentiate between DR stages with limited overlap across adjacent classes.

**Figure 17:**
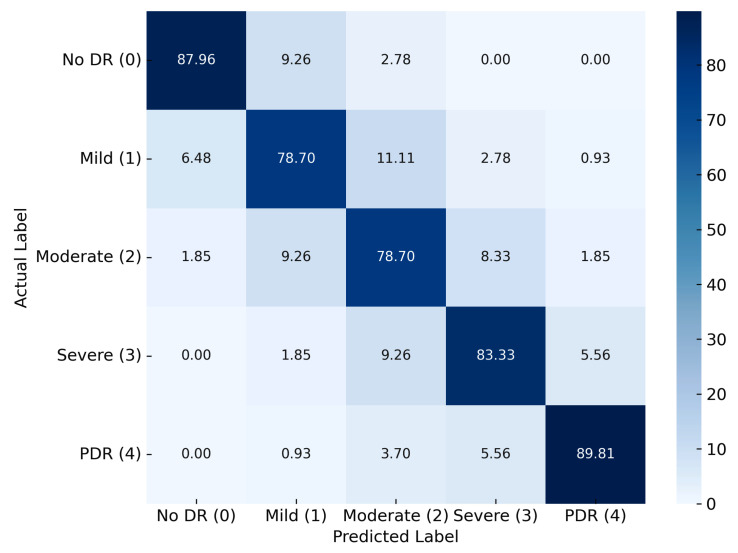
Confusion matrix for Dataset II.

The confusion matrix for Dataset II, as shown in Figure 17, processed using the same pipeline, revealed a notable decline in performance, particularly for early and intermediate stages. No DR was classified with 87.96% accuracy, while mild DR and moderate DR both achieved only 78.70%. The increased confusion between these categories suggests challenges in distinguishing subtle pathological features. PDR attained a relatively higher accuracy of 89.81%, though some misclassification into the severe DR category was evident.

**Figure 18:**
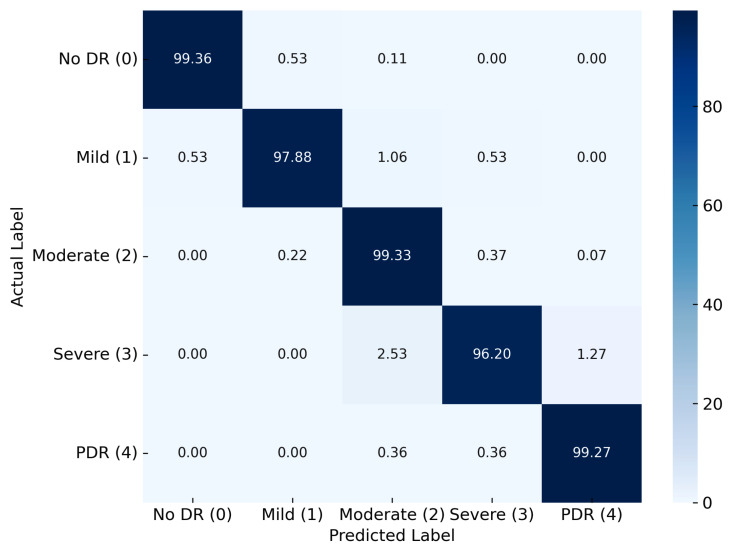
Confusion matrix for Dataset III.

The most promising results were obtained from Dataset III, using the same model configuration (Figure 18). The classification accuracy reached near-perfect levels for no DR (99.36%), moderate DR (99.33%), and PDR (99.27%). Mild DR and severe DR also demonstrated high precision with 97.88% and 96.20%, respectively. The minimal confusion across all stages highlights the model’s high discriminative capacity and the effectiveness of the integrated preprocessing and classification strategy. These findings collectively affirm the superiority of the proposed HLG-RetinaSR + RefineNet-U framework, particularly evident in Dataset III, and underscore the importance of high-quality preprocessing and advanced architecture design in achieving reliable DR stage classification.

**Figure 19:**
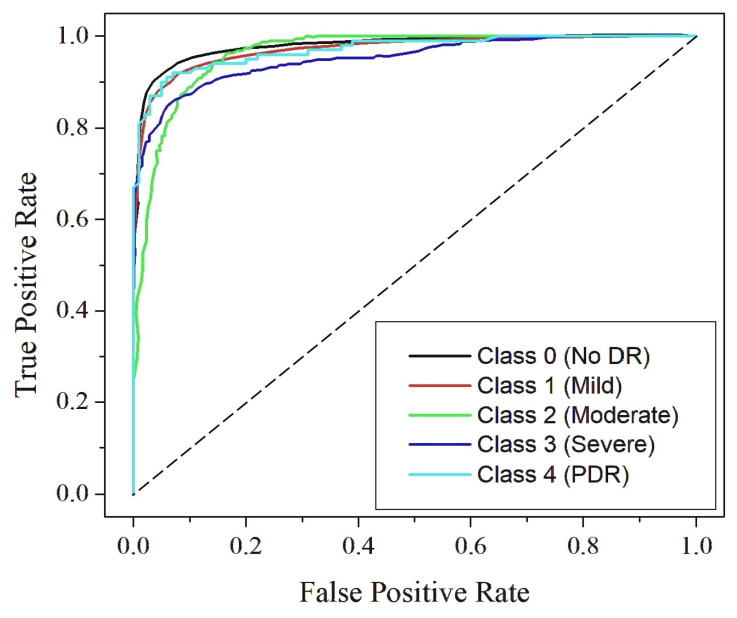
ROC curve for Dataset I.

**Figure 20:**
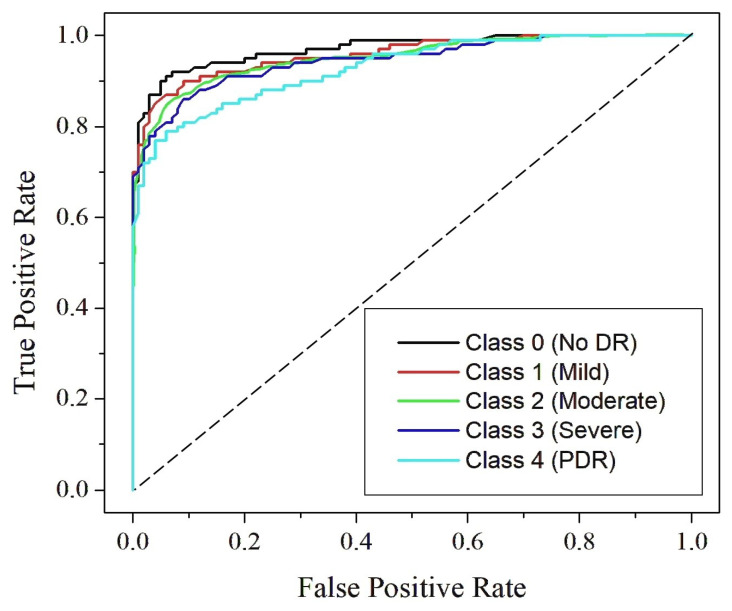
ROC curve for Dataset II.

**Figure 21:**
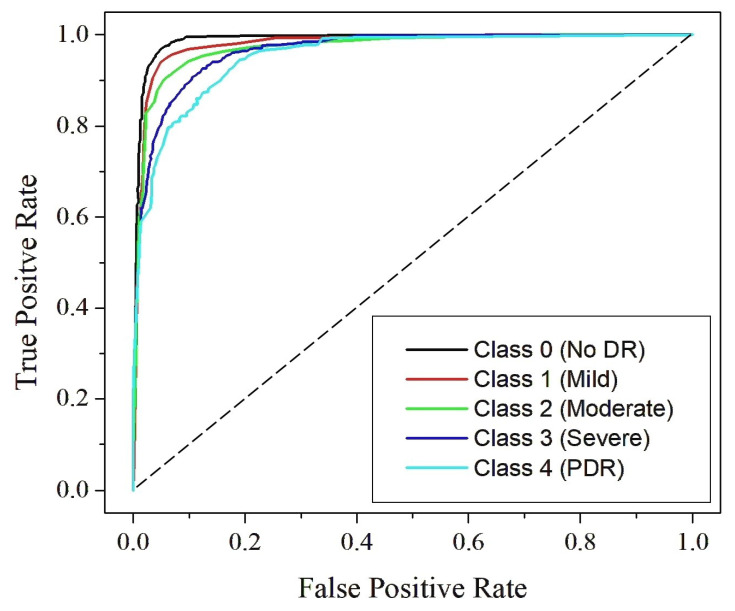
ROC curve for Dataset III.

Figure 19, Figure 20 and Figure 21 present the Receiver Operating Characteristic (ROC) curves for Dataset I, Dataset II, and Dataset III, respectively. These figures cover the five DR severity grades: Class 0 (no DR), Class 1 (mild), Class 2 (moderate), Class 3 (severe) and Class 4 (PDR). Each ROC curve illustrates the trade-off between the true positive rate and false positive rate across varying thresholds, thereby providing a measure of the model’s discriminative capability. The curve for Class 0 (no DR) lies close to the upper boundary in all datasets, reflecting reliable separation between healthy and diseased samples. For Class 1 (mild), where early manifestations are typically subtle and harder to detect, the model demonstrates consistent accuracy, indicating its effectiveness in identifying initial pathological signs. Similarly, Class 2 (moderate) shows high sensitivity, confirming the model’s ability to capture prominent lesions such as hemorrhages and microaneurysms. The ROC curves for Class 3 (severe) remain clustered near the top-left corner, demonstrating strong detection of advanced pathological symptoms, while those for Class 4 (PDR) lie well above the diagonal reference line across all datasets, highlighting the robustness of the model in recognizing the most critical stage of the disease. The computed AUC values, ranging from 0.87 to 0.99, emphasize excellent classification performance with near-perfect separation between classes. The clustering of ROC curves near the ideal boundary reinforces the reliability and strong generalization capability of RefineNet-U for multi-class DR detection.

### 4.5. Comparison with State-of-the-Art Techniques

The performance of the proposed model was thoroughly compared with that of existing models from previously published research, as shown in Table 9. The first comparison is made with different models used for DR classification on the APTOS-2019 dataset. The models listed include those from previously published research, along with the proposed model, evaluating their performance based on accuracy, recall, precision, F1-score, and AUC. Islam et al. (2022) [61] implemented a hybrid approach integrating the Xception architecture with the SCL model, which resulted in an accuracy of 84.36%, a recall of 73.84%, a precision of 70.51%, and an F1-score of 70.49%. Additionally, their method achieved an AUC value of 93.82%. In a separate study, Guo et al. (2022) [62] proposed the AABNet framework, demonstrating enhanced performance metrics in comparison to prior techniques.

The table also presents a comparative evaluation of different models used for DR classification on the DDR dataset. He et al. (2021) [66] introduced DenseNet-121 with CABNet, achieving an accuracy of 78.98%, though recall, precision, F1-score, and AUC values are not provided. Guo et al. (2022) [62] implemented AABNet, reporting an accuracy of 77.15%, recall of 58.77%, precision of 63.76%, F1-score of 59.54%, and AUC of 82.23%. Wang et al. (2022) [67] proposed Deep MT-DR, which significantly improved performance with an accuracy of 83.60%, recall of 83.1%, and F1-score of 83, though precision and AUC values are not mentioned. In comparison, the proposed model, RefineNet-U, outperforms all previous approaches by achieving an impressive accuracy of 99.11%, a recall of 99.19%, a precision of 99.17%, an F1-score of 99.12%, and an AUC of 98.97%. This demonstrates the superior effectiveness of the proposed model in accurately classifying DR cases, highlighting its robustness and reliability in medical image analysis.

## 5. Conclusions

This work presents a unified framework for multiclass DR classification by integrating a novel HLG-RetinaSR enhancement module with a customized RefineNet-U classifier. The proposed approach enhances lesion visibility and improves classification accuracy by fusing local and global image features. Experimental results confirm that the dual-stage design achieves over 99% across key performance metrics, outperforming existing methods. Unlike conventional models, this framework not only improves diagnostic precision but also enhances clinical interpretability through lesion-focused feature enhancement. Its scalable design and high reliability demonstrate strong potential for deployment in real-world DR screening applications, supporting early detection and reducing diagnostic burden.

## Figures and Tables

**Table 1:** Visual representation of retinal image against enhancement techniques obtained using SRCNN, EDSR, SwinIR, and the proposed HLG-RetinaSR model.

Original	SRCNN	EDSR	SWIN IR	HLG-RetinaSR
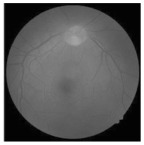	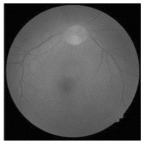	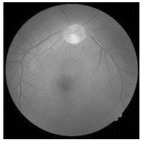	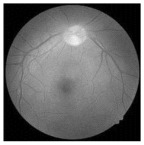	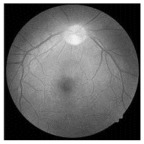
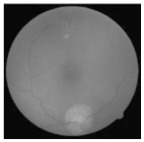	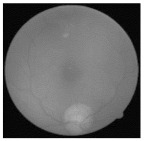	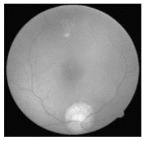	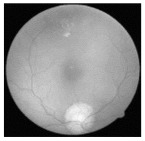	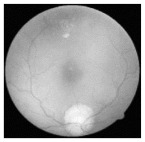
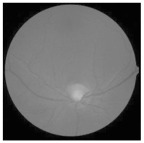	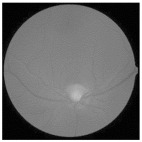	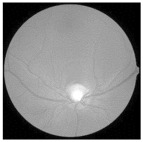	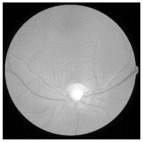	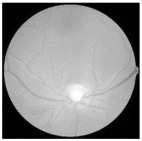

**Table 5:** Dataset description.

Dataset No.	Dataset	Number of Images	Image Size	DR Severity Levels	Hospital/Institution
I	APTOS	5590	224 × 224, 512 × 512 pixels	5	Asia Pacific Tele-Ophthalmology Society (APTOS), India
II	MESSIDOR	1800	1440 × 960 pixels		INRIA (French National Institute for Research in Computer Science and Automation)
III	DDR	13,673	Multiple sizes		Institute of Automation, Chinese Academy of Sciences (CASIA), China

**Table 6:** Performance evaluation of Dataset I.

Enh. Tech.	Class. Tech.	Train Acc (%)	Test Acc (%)	Recall	Pre. (%)	F1-Score	Sens	Spec	AUC
SRCNN	CNN	90.01	80.21	0.86	80.24	0.83	0.86	0.73	0.79
	DenseNet121	92.01	81.7	0.87	81.7	0.84	0.87	0.75	0.81
	RefineNet-U	95.87	83.47	0.88	83.48	0.86	0.88	0.77	0.83
EDSR	CNN	90.89	81.21	0.87	81.24	0.84	0.87	0.74	0.8
	DenseNet121	92.67	82.15	0.87	82.15	0.85	0.87	0.75	0.81
	RefineNet-U	94.74	84.1	0.89	84.11	0.86	0.89	0.78	0.83
Swin IR	CNN	91.88	81.16	0.87	81.19	0.84	0.87	0.74	0.8
	DenseNet121	93.87	81.97	0.87	81.98	0.85	0.87	0.75	0.81
	RefineNet-U	96.03	85.16	0.9	85.16	0.87	0.9	0.79	0.84
HLG-RetinaSR	CNN	94.04	82.9	0.88	82.92	0.85	0.88	0.76	0.82
	DenseNet121	97.1	91.32	0.94	91.35	0.93	0.94	0.88	0.91
	RefineNet-U	98.9	97.42	0.98	97.45	0.98	0.98	0.96	0.97

**Table 7:** Performance evaluation of Dataset II.

Enh. Tech.	Class. Tech.	Train Acc (%)	Test Acc (%)	Recall	Pre. (%)	F1-Score	Sens	Spec	AUC
SRCNN	CNN	82.01	70.27	0.78	70.24	0.74	0.78	0.61	0.7
	DenseNet121	87.03	73.33	0.8	73.3	0.77	0.8	0.65	0.73
	RefineNet-U	91.9	76.41	0.83	76.5	0.8	0.83	0.68	0.76
EDSR	CNN	87.04	71.87	0.79	71.91	0.75	0.79	0.63	0.71
	DenseNet121	91.07	75.77	0.82	75.8	0.79	0.82	0.68	0.75
	RefineNet-U	93.1	76.99	0.83	77.05	0.8	0.83	0.69	0.76
Swin IR	CNN	88.06	74.53	0.81	74.55	0.78	0.81	0.66	0.74
	DenseNet121	92.09	77.98	0.84	78.03	0.81	0.84	0.7	0.77
	RefineNet-U	94.05	82.22	0.87	82.2	0.85	0.87	0.75	0.81
HLG-RetinaSR	CNN	89.07	79.09	0.85	79.14	0.82	0.85	0.72	0.78
	DenseNet121	94.06	82.03	0.87	82.06	0.85	0.87	0.75	0.81
	RefineNet-U	97.89	87.66	0.91	87.76	0.9	0.91	0.83	0.87

**Table 8:** Performance evaluation of Dataset III.

Enh. Tech.	Class. Tech.	Train Acc (%)	Test Acc (%)	Recall	Pre. (%)	F1-Score	Sens	Spec	AUC
SRCNN	CNN	91.77	83.17	0.88	83.18	0.86	0.88	0.77	0.82
	DenseNet121	93.66	83.66	0.88	83.66	0.86	0.88	0.77	0.83
	RefineNet-U	96.53	84.14	0.89	84.14	0.86	0.89	0.78	0.83
EDSR	CNN	92.06	82.18	0.87	82.19	0.85	0.87	0.75	0.81
	DenseNet121	96.05	87.4	0.91	87.41	0.89	0.91	0.82	0.87
	RefineNet-U	97.13	92.23	0.95	92.23	0.93	0.95	0.89	0.92
Swin IR	CNN	94.44	90.29	0.93	90.3	0.92	0.93	0.86	0.9
	DenseNet121	98.01	92.54	0.95	92.54	0.94	0.95	0.89	0.92
	RefineNet-U	99.04	97.2	0.98	97.21	0.98	0.98	0.96	0.97
HLG-RetinaSR	CNN	96.08	85.11	0.9	85.12	0.87	0.9	0.79	0.84
	DenseNet121	98.13	89.9	0.93	89.91	0.91	0.93	0.86	0.89
	RefineNet-U	99.87	99.11	0.99	99.11	0.99	0.99	0.99	0.99

**Table 9:** Comparative analysis of proposed work with state-of-the-art techniques.

Dataset	Model	References	Accuracy	Recall	Precision	F1-Score	AUC
**APTOS-2019**	Xception and SCL	(Islam et al., 2022) [61]	84.36	73.84	70.51	70.49	93.8
	AABNet	(Guo et al., 2023) [62]	86.02	75.57	67.87	71.51	86
	Baseline with dual attention	(Bodapati & Balaji, 2024) [63]	86.22	72.23	80.61	75.41	96.5
	Capsule network and Inception	(Oulhadj et al., 2023) [64]	86.54	87	86	86	–
	CWN	(Han et al., 2023) [65]	86.12	–	–	–	–
	**Proposed Model**	**RefineNet-U**	**97.42**	98.04	97.45	97.97	97.1
**DDR**	DenseNet-121 with CABNet	(He et al., 2021) [66]	78.98	–	–	–	–
	AABNet	(Guo et al., 2022) [62]	77.15	58.77	63.76	59.54	82.2
	DeepMT-DR	(Wang et al., 2022) [67]	83.6	83.1	–	83	–
	**Proposed Model**	**RefineNet-U**	**99.11**	**99.19**	**99.17**	**99.12**	**99**

## Data Availability

All data supporting the findings of this study are publicly available from the resources cited in the article.
